# Biosensing circulating MicroRNAs in autoinflammatory skin diseases: Focus on Hidradenitis suppurativa

**DOI:** 10.3389/fgene.2024.1383452

**Published:** 2024-04-09

**Authors:** Chiara Moltrasio, Carlos André Silva, Paola Maura Tricarico, Angelo Valerio Marzano, Muhammad Sueleman, Sergio Crovella

**Affiliations:** ^1^ Dermatology Unit, Fondazione IRCCS Ca’ Granda Ospedale Maggiore Policlinico, Milan, Italy; ^2^ University Center CESMAC, Maceio, Brazil; ^3^ Department of Advanced Diagnostics, Institute for Maternal and Child Health-IRCCS Burlo Garofolo, Trieste, Italy; ^4^ Department of Pathophysiology and Transplantation, Università degli Studi di Milano, Milan, Italy; ^5^ Laboratory of Animal Research (LARC), Qatar University, Doha, Qatar

**Keywords:** Hidradenitis suppurativa, circulating miRNA, early diagnosis, therapeutic follow-up, peptides design, advanced computational tools

## Abstract

MicroRNAs (miRNAs) play a crucial role in the early diagnosis of autoinflammatory diseases, with Hidradenitis Suppurativa (HS) being a notable example. HS, an autoinflammatory skin disease affecting the pilosebaceous unit, profoundly impacts patients’ quality of life. Its hidden nature, with insidious initial symptoms and patient reluctance to seek medical consultation, often leads to a diagnostic delay of up to 7 years. Recognizing the urgency for early diagnostic tools, recent research identified significant differences in circulating miRNA expression, including miR-24-1-5p, miR-146a-5p, miR26a-5p, miR-206, miR338-3p, and miR-338-5p, between HS patients and healthy controls. These miRNAs serve as potential biomarkers for earlier disease detection. Traditional molecular biology techniques, like reverse transcription quantitative-polymerase chain reaction (RT-qPCR), are employed for their detection using specific primers and probes. Alternatively, short peptides offer a versatile and effective means for capturing miRNAs, providing specificity, ease of synthesis, stability, and multiplexing potential. In this context, we present a computational simulation pipeline designed for crafting peptide sequences that can capture circulating miRNAs in the blood of patients with autoinflammatory skin diseases, including HS. This innovative approach aims to expedite early diagnosis and enhance therapeutic follow-up, addressing the critical need for timely intervention in HS and similar conditions.

## 1 Introduction

MicroRNAs (miRNAs), characterized as small non-coding RNA molecules, play a pivotal role in cellular processes by regulating gene expression and contributing significantly to the modulation of the immune system. In the context of autoinflammatory diseases, the early stages often exhibit distinctive and aberrant miRNA expression patterns, presenting a promising avenue for potential biomarkers (Poniewierska-Baran et al., 2023). The dysregulation of miRNAs not only reflects the complex interplay within inflammatory pathways but also provides valuable insights into the underlying mechanisms of these diseases (Das and Rao, 2022). Harnessing the power of miRNA signatures allows for the identification of specific molecular profiles associated with the initial phases of autoinflammatory diseases. This capability not only enhances our understanding of disease progression but also enables clinicians to establish timely and accurate diagnostic approaches, paving the way for more effective intervention and management strategies.

In the context of skin autoinflammatory diseases, Hidradenitis suppurativa (HS) is a chronic autoinflammatory skin condition primarily impacting the terminal hair follicles. HS typically emerges post-puberty but can also occur in pediatric patients (aged <18 years): 7% of HS patients experience symptoms before age 13 and in 2% before 11 (Cotton et al., 2023). It manifests through the formation of painful, deeply entrenched nodules, abscesses, and sinus tracts, most localized in areas such as the axillary, inguinal, ano-genital, or infra-mammary regions (Sabat et al., 2020). While diagnostic criteria involve characteristic lesions, impacted sites, and the chronic nature of the condition, complications arise due to the variable presentation and the propensity of early HS lesions to mimic other skin disorders, posing challenges in accurate diagnosis (Zouboulis et al., 2020; Preda-Naumescu et al., 2021). The presence of malodorous, purulent lesions coupled with the psychological implications of diminished self-confidence can result in HS patients hesitating to seek medical consultation. This amalgamation of factors collectively contributes to an average diagnostic delay of 7–10 years (Saunte et al., 2015; Garg et al., 2020).

The impact of HS on patient quality of life is profound, and unmet needs persist. A survey conducted by the VOICE Global Survey of Impact and Healthcare Needs (VOICE) (Garg et al., 2020) underscores the fact that nearly 40% of HS patients experience significant deterioration in their quality of life due to the disease, underscoring the urgency for effective treatment solutions.

In fact, it must be considered that, to date, HS still retains its status as an “orphan disease”, with pathogenesis poorly understood and delay in diagnosis. This classification stems from its intricate etiology and the absence of unequivocal, reproducible methodologies for swift and precise diagnosis, particularly during the initial stages of the condition.

The absence of definitive biological markers capable of ensuring unambiguous and rapid early-stage disease diagnosis remains a challenge. miRNAs are small noncoding RNA molecules intricately involved in post-transcriptional gene expression regulation. Their activity has been linked to a spectrum of diseases, including autoimmune/autoinflammatory disorders. While regarded as a promising diagnostic tool, their untapped potential remains.

A recent study by De Felice et al. (De et al., 2022) employed conventional molecular techniques to reveal distinctive miRNA expression profiles, notably miR-24-1-5p, miR-146a-5p, miR26a-5p, miR-206, miR338-3p, and miR-338-5p, in HS patients compared to healthy controls. Of particular significance is the overexpression of miR-338-5p in HS lesional tissue, intricately linked to invasiveness and the secretion of pro-inflammatory cytokines; this finding led the authors to postulate its pivotal role in HS pathogenesis.

Additionally, recent studies have highlighted the involvement of a diverse range of miRNAs in regulating the phosphoinositide 3-kinase/protein kinase B/mTOR (PI3K/AKT/mTOR) signaling pathway. This regulation influences the expression and function of various components within the pathway, and as a result, the dysregulated expression of these miRNAs may impact the mTOR pathway, leading to abnormal immune responses and inflammation (Nazari et al., 2021). Specifically, the dysregulation of the mTOR pathway has been implicated in common inflammatory diseases, including HS, where it could potentially serve as a molecular marker associated with disease severity or clinical response (Soltani et al., 2018). Furthermore, Balato et al. (Balato et al., 2019) conducted an analysis on HS patients, revealing the effects of the tumor necrosis factor (TNF)-α antagonist adalimumab on mTOR Complex 1 (mTORC1) activity. This study underscores the specific involvement of mTORC1 in HS pathogenesis and suggests its potential as a novel mechanism through which TNF-α inhibition improves HS.

While the identification of miRNAs illuminates disease mechanisms, achieving rapid and cost-effective detection methods remains a hurdle. To date, the widely used method for miRNA expression is reverse transcription quantitative-polymerase chain reaction (RT-qPCR) that, using specific primers and probes, shows high sensitivity, specificity, and reproducibility. Similarly, Next-Generation Sequencing (NGS) technologies provide an opportunity to research the miRNA expression profiles in detail, but despite their utility, are often expensive and time-consuming.

Alternatively, short peptides offer a versatile and effective means for capturing miRNAs, providing specificity, ease of synthesis, stability, and multiplexing potential, allowing a rapid, efficient, and cost-effective analysis.

Peptides are short amino acid chains, linked together by peptide bonds. Short peptides can be easily synthesized on a large scale, making the development of diagnostic and therapeutic tools economically affordable. The number of different properties that can be obtained by combining the natural (or not) amino acids is potentially enormous, making the design of peptides capable of recognizing any molecule, including miRNAs. The large number of possible sequences and the intrinsic flexibility of peptides call for a computational procedure capable of screening the most valuable candidates, whose affinity toward the target is afterward tested in the laboratory, and finally used for the engineering of devices and/or therapeutic tools.

In the essence, advanced computational tools enhanced the accuracy, speed and scope of disease diagnosis, revolutionizing healthcare by enabling early intervention, personalized treatment, and a deeper understanding of disease mechanisms. Their integration into medical practice holds immense potential for improving patient outcomes and overall public health.

Here, we describe the advanced computational methods suitable for an *in silico* design of miRNA-specific peptides trying to contribute to the development of early diagnostic systems and follow-up management strategies for HS; this could enable clinicians to identify the disease for appropriate treatment, thus increasing the chances of success and consequently improving patients’ quality of life.

## 2 Computation strategy for peptide design

### 2.1 Selection, identification, and characterization of sequences

Probe sequences that harbor RNA-binding motifs will be gathered from various databases, including NCBI (National Center for Biotechnology Information), ATtRACT (Giudice et al., 2016), cisBP RNA (Ray et al., 2013), mCrossBase (Feng et al., 2019), oRNAment (Benoit Bouvrette et al., 2020), and RBPmap (Paz et al., 2014), along with manual literature searches. The identification of these motifs within the available NBCI proteomes will be carried out through mining methods such as BLAST and Hidden Markov Model, established and utilized by the proposing group.

### 2.2 Peptide design

The adopted methodology commences with the establishment of a comprehensive database comprising a diverse range of candidate peptide fragments (fragPep) of varying sizes. This database will be constructed using the PepDraw2.0 tool (GITUB, 2024), which facilitates the accurate and structurally varied generation of these fragPep. Subsequently, a meticulous analysis of the acquired fragPep will be conducted. This analysis will entail the implementation of an array of machine learning-based predictors, selected for their recognized abilities to evaluate and predict peptide-RNA binding affinity. The particulars of these predictors are detailed in <b>Table 1</b>. The resultant outcomes of the predictive analysis provided by each employed predictor will be assessed based on their cutoffs, enabling the categorization of fragPep according to their potential for RNA binding. Evaluating the results against these cutoffs is pivotal in identifying potential fragPep with RNA binding potential, representing the most promising candidates for subsequent steps.

**TABLE 1:** Traditional machine learning predictor models showing their used classifier types.

Name	Classifier	Link	References
RPISeq	SVM/RF	http://pridb.gdcb.iastate.edu/RPISeq/	Ray et al. (2013)
catRAPID	SVM	http://service.tartaglialab.com/update_submission/731665/ea01402cd6	Feng et al. (2019)
RPI-Pred	SVM/RF/KNN/NB/DT	http://ctsb.is.wfubmc.edu/projects/rpi-pred	Benoit Bouvrette et al. (2020)
RNApred	SVM	https://webs.iiitd.edu.in/raghava/rnapred/submit.html	Paz et al. (2014)
Pprint	SVM	https://webs.iiitd.edu.in/raghava/pprint/submit.html	GITUB (2024)
RNABindRPlus	SVM	https://bio.tools/rnabindrplus	Varadi et al. (2022)
PRIdictor	SVM	http://www.rnainter.org/PRIdictor/	Prosa (2024)
PRNA	RF	https://doc.aporc.org/wiki/PRNA	Saves MBI (2024)

Support Vector Machine (SVM), Random Forest (RF), K-Nearest Neighbors (K-NN), Naive Bayes (NB), Decision Tree (DT).

### 2.3 Three-dimensional modeling and structural validation

The selected peptide sequences will undergo a detailed analysis of their three-dimensional structure, using the tool AlphaFold 2.0 (Varadi et al., 2022). AlphaFold 2.0 is an advanced protein folding prediction system developed by DeepMind, an artificial intelligence research lab and employs deep learning techniques to predict the 3D structure of proteins based on their amino acid sequences. The quality of the generated models will be submitted to a careful validation through multiple parameters, including the Z-score, the Ramachandran graph, and the Qmean DisCo. These careful evaluations aim to guarantee the quality and reliability of the predicted three-dimensional structures. The Z-score, generated by ProSA-web server (Prosa, 2024), is a statistical measurement that assesses how unusual the total energy of a protein structure is compared to structures of similar size and amino acid composition. A high z-score suggests that the structure might have significant errors or inaccuracies, while a low z-score indicates that the structure is within the range of expected energies for a protein of its size and composition. ProSA-web provides a graphical representation of the Z-score along with a color-coded visualization of local model quality, helping researchers to identify potential problems or errors in their protein structures and prioritize areas for refinement or further investigation. Ramachandran graph (Saves MBI, 2024) allows the structure of proteins to be confirmed, by analyzing the conformation of the dihedral angles of the protein and verifying the viability of the conformations adopted by the amino acidic residues (Laskowski et al., 1993; Gopalakrishnan et al., 2007). More in detail, Ramachandran plot is a graphical representation of the φ (phi) and ψ (psi) torsion angles of amino acid residues in a protein structure; it helps in evaluating the stereochemical quality of protein structures by showing allowed and disallowed regions for these torsion angles based on empirical observations. In a Ramachandran plot, the most favoured regions are those where the φ and ψ angles fall within certain ranges typical for secondary structure elements such as alpha helices and beta sheets. The plot also indicates outliers, which are residues with torsion angles that deviate significantly from the favoured regions, potentially indicating errors in the structure (Laskowski et al., 1993; Gopalakrishnan et al., 2007). In addition, Qmean DisCo evaluates the overall quality of the model, taking into account the degree of deviation from the native structure. These multiple combined assessments guarantee a comprehensive and detailed analysis of the conformation and stability of the theoretical models generated by AlphaFold2. With these strict quality controls, we seek to ensure that the three-dimensional structures of the designed peptides are highly reliable and ready for future phases of target miRNA binding studies.

### 2.4 Molecular docking

First, we will compile a comprehensive list of suitable miRNAs associated with HS from existing literature sources. The process of molecular docking of peptides designed with miRNAs will be conducted through the ClusPro server (Kozakov et al., 2017), a highly reliable and widely used platform for the analysis of biomolecular interactions. ClusPro is a tool that performs semi-rigid molecular anchoring, favoring the formation of bonds between molecules in an energetically favorable way. This method allows the exploration of multiple conformations of the peptides and miRNAs involved, enabling the identification of more stable geometries with greater affinity. The inherent flexibility of the ClusPro docking process is particularly useful for understanding the dynamic interactions between peptides and target miRNAs, ensuring a comprehensive and accurate analysis of possible binding conformations. The server provides the ten best complex models ranked by low energy score as the output of the docking simulation. The use of ClusPro in the present study aims to provide valuable information about the complexes formed, in order to select the most promising peptides with therapeutic potential for future experimental investigations. Our criterion for selecting the most promising miRNA-peptide pairs will hinge upon achieving high docking scores and establishing robust bonding networks, notably through hydrogen bonds, pi-pi interactions, and salt bridges. To visualize the binding interface in terms of hydrogen bond, salt bridge and non-bonded contacts between the designed peptides and target miRNA, we will use the PDBsum online server (Mohammad et al., 2021; EBI, 2024). With this robust and detailed approach, we aim to maximize the chances of success in identifying candidate peptides with high affinity and specificity for the miRNAs of interest. To ensure the accuracy and stability of molecular docking outcomes, we will employ molecular dynamic simulations, as described in detail below.

### 2.5 Dynamic stability analysis of peptides-miRNAs complexes

For the detailed study of peptide-miRNA complexes, we will employ molecular dynamics simulations, a powerful computational approach to investigate the behavior and interactions of molecules at the atomic scale. To carry out the simulations, we will use the GROMACS 2020.1 package (Abraham et al., 2015), which is widely recognized for its efficiency and accuracy in conducting molecular dynamics simulations. The GROMOS96 53A6 force field will be adopted to describe the interactions between atoms in the systems under study, while the simple point charge (SPC) water model will be used to represent the aqueous environment. The system will be properly prepared, with solvation using NaCl ions at physiological concentration (0.15M), in order to mimic the conditions of a relevant biological environment. Then, the system will be subjected to an energy minimization step, aiming to find the most stable and stress-free conformation. For the dynamic simulations, the temperature will be kept constant at 300 K and the pressure will be adjusted to 1 atm, guaranteeing thermodynamic conditions close to physiological ones.

Molecular dynamics will be conducted for a specific period, generally considering a time of 100 ns to allow molecules to explore different conformations and interactions. At the end of the simulations, the models obtained will be carefully evaluated for their three-dimensional structure and secondary structure. Detailed analyzes will be carried out to verify the stability of peptide-miRNA interactions, as well as the maintenance of structures over the simulation time. The post simulation analysis will be performed by using different packages of GROMACS 2020.1. These packages will be utilized to examine the dynamic stability, residual fluctuation, compactness, and hydrogen bonding network of the peptides-miRNA complexes. To assess the structural dynamic stability, the Root Mean Square Deviation (RMSD) will be computed. However, the Rg (radius of gyration) will be employed to calculate the structural compactness. On the other hand, to analyze the fluctuation at residues level we will calculate the Root Mean Square Fluctuation (RMSF). This comprehensive evaluation will ensure the reliability of the results obtained, enabling a better understanding of the interactions between peptides and miRNAs, and providing crucial information for the development of future therapeutic strategies.

### 2.6 Binding free energies calculation by using MM/GBSA approach

Binding free energy calculations are frequently utilized to precisely evaluate the strength of binding and structural aspects of small molecules. This calculation is of paramount importance in refining the accuracy and reliability of docking predictions, surpassing traditional docking and alchemical methods (Khan et al., 2022). This approach is extensively applied to explore the strength of interactions and unveil essential binding characteristics that dictate the comprehensive binding mechanism. Consequently, we will use the MM/GBSA methodology to assess the total binding energy of peptides-miRNA complexes (Chen et al., 2018). This involve determining the contributions of van der Waals energy (vdW), electrostatic energy, and generalized Born surface area (GB) to the total binding free energy.

**FIGURE 1:**
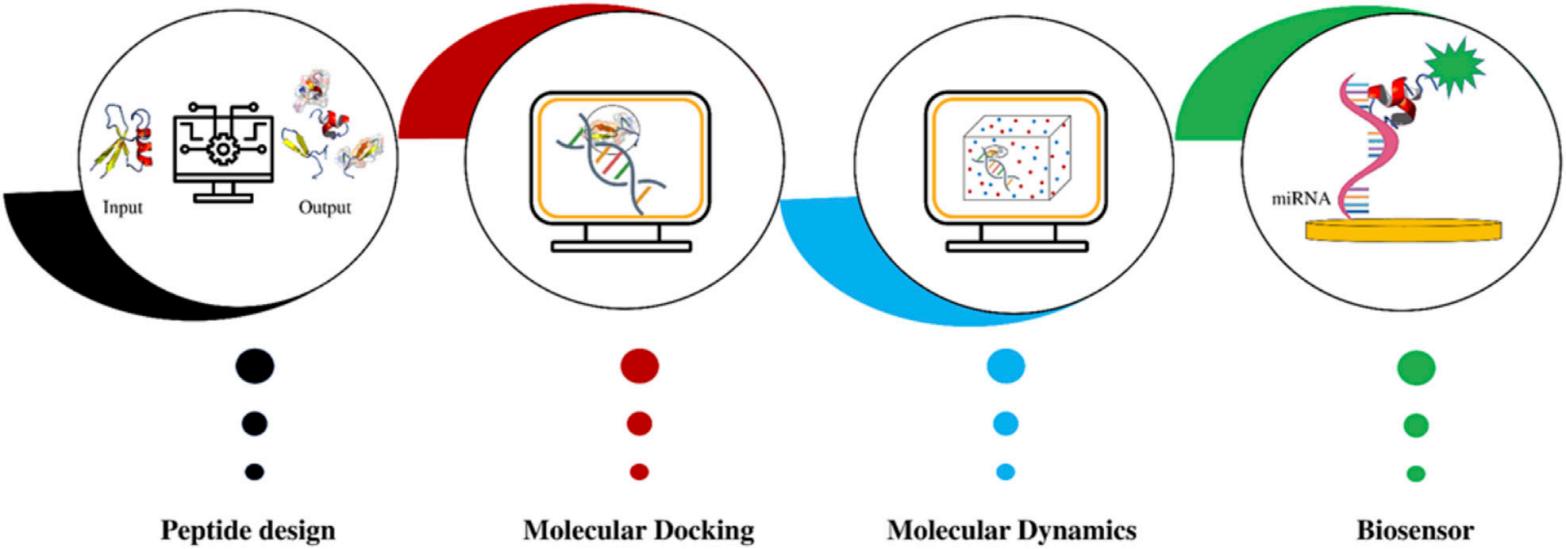
The bioinformatics pipeline for the design of peptides that specifically recognize and capture circulating miRNAs: after peptide design, molecular docking and molecular dynamics are performed; at the end, the peptide with the best affinity for the circulating miRNA will potentially be used to develop a biosensor.

The summary of the different steps for miRNA targeting peptides design is reported in <b>Figure 1</b>.

## 3 Harnessing peptide-based biosensors for miRNA capture and detection

The approach outlined above is applicable to the detection of any miRNA associated with autoinflammatory diseases using specific peptides. In this context, our emphasis will be on HS.

Once the miRNA HS specific peptides would have been designed using our computational pipeline, we will need to set-up a fast and reliable detection systems translatable to the health system with easy use, thus not needing at this stage complex laboratory procedures or molecular biology specialized personnel. This is an ambitious goal that can be achieved by using appropriate biosensors that should be designed accordingly. Biosensors are innovative tools that combine biological recognition elements with transducing components to detect and quantify specific molecules within complex samples (Yuan et al., 2023). The development of biosensors has revolutionized various fields, including medical diagnostics, environmental monitoring, and biotechnology (Escobar et al., 2023). In recent years, the integration of peptides as capturing agents in biosensors has emerged as a promising strategy for the sensitive and specific detection of miRNAs, small non-coding RNA molecules with significant regulatory roles in gene expression (Vanova et al., 2021).

### 3.1 Peptides as elements for selective recognition

Peptides, consisting of short amino acid chains, can be manipulated to demonstrate elevated specificity and affinity toward target molecules. Through meticulous design and selection of peptide sequences, researchers can craft recognition elements tailored to bind with particular miRNAs. These peptide-based recognition elements present numerous advantages compared to conventional capture probes like antibodies or nucleic acids. The synthesis of peptides allows for meticulous control over their sequence, facilitating the customization of binding properties for diverse miRNA targets.

### 3.2 Construction of biosensors using peptides

The construction of a peptide-based biosensor involves several steps:

1. Peptide Selection: Computational methods, such as phage display or combinatorial peptide libraries, are used to identify peptides with high affinity and selectivity for the target miRNA.
2. Surface Immobilization: The selected peptide is immobilized onto the biosensor surface, which can be a variety of materials, such as silicon chips, nanoparticles, or electrode surfaces.
3. Transduction Mechanism: The interaction between the peptide and target miRNA leads to a measurable signal. This signal can be based on optical, electrical, or mass-based changes, depending on the transduction method employed.
4. Signal Amplification: To enhance sensitivity, signal amplification strategies, such as enzyme labeling or nanoparticle-based amplification, can be incorporated into the biosensor design.

### 3.3 Benefits of using peptide-based biosensors for miRNA detection

1. Specificity: Peptides can be designed to specifically recognize unique sequences in target miRNAs, minimizing cross-reactivity and false positives.
2. Customizability: Peptide sequences can be easily manipulated to optimize binding affinity and selectivity for different miRNA targets.
3. Stability: Peptides can exhibit better stability under various conditions compared to other recognition elements like antibodies.
4. Non-Invasiveness: Peptide-based biosensors often allow for non-invasive sample collection, reducing the need for invasive procedures, even if in our case we will just need a blood sample due to the circulating nature of the targeted miRNA.
5. Ease of Synthesis: Peptides can be chemically synthesized with high reproducibility and scalability.

### 3.4 Challenges and outlook for the future

Although the potential of peptide-based biosensors is promising, there are persistent challenges in terms of stability, reproducibility, and validation that need attention. When the target miRNA is present in a sample, it binds to the peptide, triggering a detectable change in the biosensor’s properties. This change can be electrical, optical, or mechanical, depending on the biosensor’s design, and is measured to indicate the presence and, in some cases, the concentration of the target miRNA in the sample. However, subsequent research is expected to concentrate on enhancing biosensor design, refining detection techniques, and broadening the scope of accurately detectable miRNA targets. The incorporation of peptides as capture agents in biosensors represents a robust strategy for the precise and sensitive detection of miRNAs. This innovative technology holds significant promise in advancing our comprehension of miRNA functions, understanding disease mechanisms, and enabling personalized diagnostics. Ultimately, this contributes to advancements in healthcare and various biotechnological applications.

## 4 Discussion

MiRNAs, small non-coding RNA molecules around 22 nucleotides in length, play a crucial role in governing post-transcriptional gene regulation. They achieve this by binding to complementary sequences in the 3′untranslated regions of target messenger RNA (mRNA), leading to mRNA degradation or translational hindrance. These miRNAs influence diverse biological processes, including cell differentiation, proliferation, apoptosis, and immune responses. Disruptions in genes targeted by miRNAs have been linked to various diseases, including autoimmune and autoinflammatory skin disorders. Psoriasis serves as an illustrative example, where dysregulation in miRNA expression profiles affects keratinocyte proliferation, differentiation, and inflammation (Moltrasio et al., 2022). Moreover, miRNAs impact the function of immune cells involved in the pathogenesis of psoriasis, such as CD4^+^ T cells, regulatory T (Treg) cells, dendritic cells, and Langerhans cells (Jiang et al., 2023). This has prompted exploration of these miRNAs as potential therapeutic targets, with ongoing preclinical studies showing promise in psoriasis treatment using exogenous miRNAs or anti-miRNA agents. However, challenges persist, necessitating further research and resolution (Jiang et al., 2023).

HS is characterized by chronic inflammation and immune dysregulation, thereby establishing a fertile ground for the exploration of miRNA involvement. Multiple studies have unveiled altered miRNA expression profiles in HS-affected skin and blood, underscoring their potential as diagnostic markers. The transformative capacity of miRNAs encompasses the modulation of genes underpinning inflammation, immune responses, and tissue remodeling—factors pivotal to HS pathogenesis (Hessam et al., 2017; Radhakrishna et al., 2022).

The challenges in early detection of HS stem from its diverse clinical presentation and the absence of specific diagnostic assays. MiRNAs emerge as promising biomarkers for early detection, given their robust presence in various biological fluids like blood, serum, and saliva. The convenience of collecting miRNAs from these fluids allows for non-invasive diagnostic approaches. Identifying specific miRNA expression patterns associated with HS has the potential to distinguish individuals at risk or in the early stages of the condition, enabling timely intervention and improved disease management.

As mentioned-above, various circulating miRNAs exhibit dysregulation in both HS-affected tissues and blood (Wang et al., 2016). These miRNAs are recognized for their interactions with genes embedded within inflammatory pathways, encompassing Toll-like receptor signaling, cytokine production, and immune cell recruitment (Borgia et al., 2022). As a consequence, it can be hypothesized that their dysregulation perpetuates HS-related inflammation and serves as indicative biomarkers for diagnosis.

Despite recent strides, notably in the work of De Felice et al. (De et al., 2022), the exploration of miRNAs within the clinical framework of HS remains at its preliminary stages. While the potential of miRNAs as early detection biomarkers for HS is promising, there are challenges to overcome. Standardization of sample collection, miRNA extraction, and quantification methods is essential to ensure robust and reproducible results. Additionally, further research is needed to validate candidate miRNAs in larger and diverse cohorts and to elucidate the mechanisms underlying their dysregulation in HS.

All in all, miRNAs could represent a novel avenue for the early detection of HS.

By identifying specific miRNA expression patterns associated with HS, researchers and clinicians can potentially revolutionize early detection strategies, leading to improved patient outcomes and better clinical and follow-up management of this challenging skin disorder.

In this landscape, the emergence of *in silico* designed peptides has opened up novel avenues for miRNA detection, offering a promising approach that combines computational prowess with biological insight (Cadoni et al., 2020; Wang et al., 2022). Peptides, short chains of amino acids connected by peptide bonds, can be meticulously designed, as we described in the methods section, to exhibit high affinity and specificity for target molecules. Leveraging this design potential, *in silico* methods enable the creation of peptides with a tailored capacity to recognize specific miRNA sequences. This approach presents a host of advantages, including cost-effectiveness, rapid development, and the potential for non-invasive diagnostic applications.

One of the critical challenges in miRNA detection lies in achieving specificity and sensitivity. Conventional methods often grapple with issues related to cross-reactivity and interference from similar sequences. In silico designed peptides circumvent these hurdles by allowing precise customization of peptide sequences to uniquely match the targeted miRNA. The computational predictions guide the selection of peptides with the highest likelihood of binding to the miRNA of interest, enhancing the accuracy of detection.

Moreover, the use of *in silico* designed peptides introduces a dimension of flexibility and adaptability. As our understanding of miRNA sequences and functions deepens, the peptide designs can be quickly adjusted to accommodate emerging insights. This dynamic nature of the approach ensures that miRNA detection strategies remain relevant and up to date, paving the way for potential applications in personalized medicine.

Beyond diagnostic potential, *in silico* designed peptides hold promise for therapeutic interventions. The same peptides that are engineered to detect specific miRNAs can also be harnessed to modulate their activity. By strategically interfering with dysregulated miRNAs, these peptides could potentially restore normal cellular processes, offering a new avenue for therapeutic development.

## 5 Conclusion

The potential of circulating miRNAs in the early diagnosis of autoinflammatory diseases, particularly HS, holds significant promise for reshaping the landscape of disease detection and management. The unique advantage of non-invasive monitoring through miRNAs released by affected tissues into the bloodstream presents a groundbreaking opportunity to revolutionize HS diagnostics. The distinctive expression profiles of miRNAs associated with HS, along with their stability and presence in various biological fluids, position them as potential diagnostic biomarkers. This advancement not only enables early detection but also addresses the significant diagnostic delay characteristic of HS. In this context, the innovative approach of designing miRNA-specific capturing peptides through computational methods becomes foundational. The fusion of computational biology and molecular design techniques empowers the creation of peptides with high affinity and specificity for target miRNAs. These peptides, meticulously engineered to recognize and bind to disease-associated miRNAs, offer a promising avenue for accurate and rapid HS diagnosis. The potential to identify specific miRNAs indicating disease presence or progression can lead to timely interventions, minimizing the impact of the disease on patients’ quality of life. Furthermore, the utility of *in silico* designed peptides could extend beyond diagnosis, as these engineered molecules may also serve as therapeutic tools. By capturing and modulating disease-associated miRNAs, these peptides could potentially influence underlying molecular mechanisms, providing a novel avenue for therapeutic intervention. The ability to simultaneously diagnose and treat HS using a single platform showcases the transformative potential of this approach in addressing unmet clinical needs. However, it is essential to acknowledge the challenges ahead. Translating computational predictions into effective real-world applications requires rigorous validation and refinement. The intricate interplay of miRNAs and peptides within complex biological systems demands careful consideration. As these technologies transition from laboratory settings to clinical practice, collaboration between computational biologists, bioengineers, clinicians, and other experts becomes crucial to ensure the reliability, accuracy, and safety of miRNA capturing peptides.

The integration of circulating miRNAs and the computational design of capturing peptides signifies a paradigm shift in HS diagnosis and management.

In clinical practice, this approach could revolutionize HS management. For instance, patients at risk of developing HS could undergo screening for specific miRNA markers, enabling early diagnosis and the commencement of pre-emptive treatments. Moreover, monitoring changes in miRNA levels could help assess the efficacy of prescribed therapies, allowing clinicians to adjust treatment plans in real-time based on molecular responses.

Despite its promise, this innovative approach faces several challenges. The specificity and sensitivity of miRNA-based diagnostics need to be optimized to distinguish between HS and other inflammatory conditions accurately. Furthermore, the development of peptide-based biosensors must overcome hurdles related to stability, biocompatibility, and the potential for immune responses. Additionally, translating these technologies from the laboratory to the bedside involves navigating regulatory pathways, demonstrating clinical validity and utility, and ensuring cost-effectiveness.

As research progresses and these challenges are addressed, the integration of miRNA detection and peptide biosensors is poised to transform HS diagnosis and treatment. By advancing our understanding of HS at the molecular level and developing tools for early detection and personalized intervention, we can significantly improve the prognosis and quality of life for individuals affected by this debilitating condition.

## Data Availability

The original contributions presented in the study are included in the article/Supplementary material, further inquiries can be directed to the corresponding author.
